# The association between IQ in adolescence and a range of health outcomes at 40 in the 1979 US National Longitudinal Study of Youth

**DOI:** 10.1016/j.intell.2008.12.002

**Published:** 2009-11

**Authors:** Geoff Der, G. David Batty, Ian J. Deary

**Affiliations:** a MRC Social and Public Health Sciences Unit, University of Glasgow, Glasgow, UK; b MRC Centre for Cognitive Ageing and Cognitive Epidemiology, Department of Psychology, University of Edinburgh, Edinburgh, UK

**Keywords:** Intelligence, Health, Cognitive epidemiology

## Abstract

A link between pre-morbid intelligence and all cause mortality is becoming well established, but the aetiology of the association is not understood. Less is known about links with cause specific mortality and with morbidity. The aim of this study is to examine the association between intelligence measured in adolescence and a broad range of health outcomes ascertained at 40 years of age. We use data on 7476 participants in the US National Longitudinal Survey of Youth 1979 who had their cognitive ability measured at baseline and completed the ‘Health at 40’ interview module between 1998 and 2004. The Health at 40 module includes assessments of general health and depression, nine medically diagnosed conditions, and 33 common health problems. Higher mental test scores were associated with lower depression scores, better general health, significantly lower odds of having five of the nine diagnosed conditions and 15 of the 33 health problems. A health disadvantage of higher cognitive ability was evident for only three of the 33 health problems.

## 1 Introduction

The beginnings of cognitive epidemiology can perhaps be traced to O'Toole and Stankov's (1992) finding an association between general intelligence and mid-life mortality in male Australian Vietnam veterans, and Whalley and Deary's (2001) demonstration that age 11 IQ predicted survival to age 76 in a Scottish population. Prior to that, research on the association between cognition and health had been primarily focused on the opposite causal direction, whereby poor health impairs cognitive functioning. A link between mental ability at age 11 and deaths over the following six and a half decades immediately suggested the possibility that intelligence differences might have a causal effect on longevity and, by implication, on health. A systematic review (Batty, Deary, & Gottfredson, 2007) and more recently published work (Batty, Shipley, Mortensen, Boyle et al., 2008; Pearce, Deary, Young, & Parker, 2006) have confirmed the link for all cause mortality. However, all cause mortality is relatively uninformative for aetiology, mechanisms and pathways. For cause specific mortality the information is sparser and more mixed.

A number of studies have reported an association between early cognition and cardiovascular, or coronary heart disease, mortality (Batty, Der, Macintyre, & Deary, 2006; Batty, Shipley, Mortensen, Gale, & Deary, 2008; Osler et al., 2003; Pavlik et al., 2003). There is some evidence for an association with lung and stomach cancer in Scotland (Deary, Whalley, & Starr, 2003; Hart et al., 2003) but not in Sweden (Batty, Modig Wennerstad et al., 2007). In that study, almost a million men were followed up to early middle age, but there was no association between intelligence at conscription and any of a large number of cancers, except for skin cancer which was associated with higher intelligence. Among external causes of death there is also a mixed picture: lower intelligence test scores are associated with death by suicide (Gunnell, Magnusson, & Rasmussen, 2005) and homicide (Batty, Deary, Tengstrom, & Rasmussen, 2008; Batty, Mortensen, Gale, & Deary, 2008) and to a combination of injuries and suicides (Osler et al., 2003).

Studies that have examined the relationship of intelligence to the risk factors for cardiovascular and respiratory disease have found that higher cognitive test scores are associated with lower rates of smoking and higher smoking cessation; lower likelihood of being overweight or obese; less heavy alcohol consumption and less hypertension (Batty, Deary, & Macintyre, 2006; Batty, Deary, & Macintyre, 2007; Batty, Deary, Schoon, & Gale, 2007; Chandola, Deary, Blane, & Batty, 2006; Starr et al., 2004; Taylor et al., 2003), although one study found an association with greater levels of problem drinking(Batty, Deary et al., 2008).

In summary, there are far fewer studies of the association between early life intelligence and later morbidity than there are for mortality. Such studies, if they can identify which health outcomes are associated with prior ability, can be of assistance in identifying the causal paths between early life intelligence and survival. For example, hypertension, diabetes and high cholesterol are all risk factors for cardiovascular disease. Differing patterns of association with intelligence might indicate pathways that link intelligence with later mortality. Studies showing link with morbidity, rather than mortality, are principally concerned with cardiovascular disease (Hart et al., 2004; Hemmingsson, v Essen, Melin, Allebeck, & Lundberg, 2007) and psychiatric disorders (Batty, Mortensen, & Osler, 2005; Walker, McConville, Hunter, Deary, & Whalley, 2002; Zammit et al., 2004). The literature showing links with morbidity is also the most likely to contain publication bias. Finding that intelligence is associated with a specific disease is much more likely to lead to a publication than an equivalent null finding.

Beyond cardiovascular and psychiatric illness, little is known about the association of early IQ with health more generally. Using data from the 1932 Scottish Mental Survey, Starr and colleagues (Starr, Deary, Lemmon, & Whalley, 2000) examined the relationship between IQ at age 11 and health status at age 77. Of 12 disease categories (including ‘other’) only dementia was associated with childhood IQ, although survivor bias may have diluted the effects. There was a positive association with functional independence, as measured by the Barthel score (Wade & Collin, 1988). Martin and colleagues (2004) looked at IQ, aged seven, and eight diagnosed conditions at age 30–39. Although the sample size was only large enough to formally analyze the association of IQ with having any of the conditions and with the total number of conditions, the authors concluded that the association “appeared to be general and not limited to a specific illness”.

There is clearly a need within cognitive epidemiology for studies which examine the relationship of early life intelligence to general health in mid life with a large enough sample size to detect a range of possible associations. The present study aims to do this in a large, population based sample, with a wide variety of health outcomes.

## 2 Method

### 2.1 Participants

The data are derived from the U.S. National Longitudinal Survey of Youth 1979 (NLSY79) (Center for Human Resource Research, 2004). This is a population representative sample of 12,686 young people who were aged 14 to 21 on 31st December 1978. Households were sampled and all inhabitants in the target age range included. Of the 8770 households, 2862 included more than one respondent. The respondents were first interviewed in 1979 and were re-interviewed annually until 1994 and biennially thereafter. From 1998 an extended health module was administered to respondents aged 40 and over. This comprises four parts: (i) a 7 item version of the Center for Epidemiological Studies Depression Scale (CES-D) (Radloff, 1977; Ross & Mirowsky, 1989); (ii) a number of questions about contact with health professionals and the health of the subject's parents; (iii) the SF12 (version 1), a brief health questionnaire comprising scales for overall mental and physical health (Ware, Kosinski, & Keller, 1996); and (iv) an extensive list of health conditions. The health at 40 module was repeated in 2000, 2002 and 2004 for those aged 40+ who had not previously completed it. Here we analyze the scores from the CES-D and SF12, and data on the health conditions.

### 2.2 Measures

#### 2.2.1 Intelligence

At baseline participants were administered the Armed Services Vocational Aptitude Battery (ASVAB) which has 10 subtests: science, arithmetic, word knowledge, paragraph comprehension, numerical operations, coding speed, auto and shop information, mathematics knowledge, mechanical comprehension, and electronics information.

As our measure of intelligence we used the 1989 revision of the Armed Forces Qualification Test (AFQT) which is derived from the four ASVAB subtests that are the most general and less vocationally-specific, namely: arithmetic, word knowledge, paragraph comprehension, and mathematics knowledge.^1^ The AFQT percentile score was *z* transformed to zero mean and unit SD.

### 2.3 Health outcomes

The 7 items of the CES-D are each scored 0 to 3 and the scores summed and z transformed. The SF12 is available pre-scored according to the manual (Ware, Kosinski, & Keller, 1995) as two components, physical and mental (Ware et al., 1996), with higher scores indicating better health. These scores were also z transformed. One of the items in the SF12, which forms part of the physical component, is self-assessed health (“In general, would you say your health is…. Excellent / Very Good / Good / Fair / Poor?”). As this item is a widely reported measure of health with good external and predictive validity, we have analyzed the responses to this question separately, taking fair or poor health to be a health problem.

The section on health conditions begins with a series of nine questions of the form “Has a doctor ever told you that you have …?” A positive response is followed by a number of supplementary questions adding detail such as the date of diagnosis and whether the condition is current. We have included only the response to the initial question, which we refer to as “diagnosed conditions”. Following these nine questions there are a further 33 questions of the form: “Do you have any of the following health problems (other than problems discussed earlier) ….?” These all have a simple yes/no answer and we refer to these as “self reported health problems”. The wording of the questions used to elicit the diagnosed conditions and self reported problems is given in Appendix A.

### 2.4 Control variables

There is little evidence available on which to base the choice of control variables, partly because our study includes a wider range of health outcomes than prior studies relating early IQ to later health, but also because evidence about possible pathways, mediators and moderators is scarce even for the outcomes that have been studied. Some potential confounders which exert their influence over a range of health outcomes – such as income, education and socio-economic status (SES) – are problematic as they may mediate the relationship between early intelligence and later health. Consequently, we have kept control variables to minimum. Sex, age at baseline interview and age at the later interview are included. In addition, in separate models, we used the z transformed parental socioeconomic status index derived by Herrnstein and Murray – a composite measure based on parental income, education and occupational status (Herrnstein & Murray, 1994). However, effect estimates adjusted for parental SES will be conservative because the genetically inherited component of parental intelligence also influences the parents' SES (Rowe, Vesterdal, & Rodgers, 1998).

### 2.5 Statistical analyses

Mixed Linear regression models were used for the continuous scores and mixed logistic models for the dichotomous health conditions. Random effects were included to allow for household clustering in the sample. Analyses were carried out using proc mixed and proc glimmix within SAS version 9.2.

## 3 Results

The NLSY 1979 baseline sample of 12,686 represents an 87% response rate from an initial screening of households to identify those with young people in the target age range (14–21). Between 1998 and 2004 there were 7,846 of the NLSY participants who completed the Health at 40 module. Those who completed the Health at 40 module had significantly lower AFQT scores (− 0.15 SD) than those who did not, less education (10.4 vs 10.7 years) but there was no difference in parental SES. Men were less likely to complete the health at 40 module than women (60% vs 64%).

AFQT and SES data were missing for 369 of the 7846 and SES data for another 1. The analyses are based on a sample of 7476 with complete data on AFQT, SES, age and sex. Small numbers of missing data for the individual health outcomes result in minor variations in the sample size. The working sample had a mean age at baseline of 17.9 years and at completion of the Health at 40 module of 40.6 years (SDs were 2.1 and 0.8 years, respectively). Forty eight percent were male. Nineteen percent were Hispanic, 31% black and 50% non-black, non-Hispanic.

The results of regressing the health scores on the AFQT score are shown in Table 1. Two sets of results are shown, the first are adjusted for age and sex and the second for parental SES as well. One standard deviation advantage in AFQT is associated with around a fifth of a standard deviation lower CES-D score. The SF12 physical and mental health scales are scored so that a higher score indicates better health and here one standard deviation advantage in AFQT is associated with a sixth of a standard deviation higher physical health score but only a fifteenth for the mental health score. Adjusting for parental SES attenuates the relationship with the SF12 Physical component somewhat.

As the remaining outcomes are binary, indicating the presence or absence of a health problem, the results are expressed as odds ratios together with their 95% confidence limits. The odds ratios themselves give the proportionate increase or decrease in odds of having a health problem for one standard deviation advantage in AFQT score. Values less than one indicate lower odds of a health problem for higher AFQT and those above one the converse. Where the 95% confidence interval crosses one, the odds ratio is not significantly different from one, that is, there is no significant advantage or disadvantage associated with AFQT score.

For self assessed general health (the question from the SF12), the odds ratio was 0.49 (95% CI 0.47 to 0.52, *p* < .0001) indicating that one standard deviation advantage in AFQT was associated with a halving of the odds of reporting fair or poor health. Adjustment for parental SES attenuated this slightly (OR 0.54 95% CI 0.51 to 0.57).

**Fig. 1:**
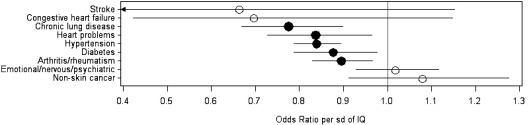
Odds ratios (with bars representing 95% confidence intervals) for a diagnosed health problem per SD of AFQT score – age adjusted. Filled circles indicate odds ratios significantly different from 1.

The results of logistic regressions of the nine diagnosed conditions on AFQT are summarized in Fig. 1 as a plot of the odds ratios and their 95% confidence intervals. In five of the nine conditions the odds of a diagnosed health problem are significantly lower with higher AFQT: chronic lung disease, heart problems, hypertension, diabetes and arthritis/rheumatism. Stroke and congestive heart failure are rare at these ages (only 20 cases of each) so that the confidence intervals are very wide. None of the odds ratios are significantly greater than one: that is, there is no suggestion that higher cognitive ability is associated with greater likelihood of having any of the health problems. The numerical results are given in Table 2. These show that adjusting for parental SES attenuates the odds ratios somewhat (that is, it brings them closer to one) and those for diabetes and heart problems were no longer significant.

**Fig. 2:**
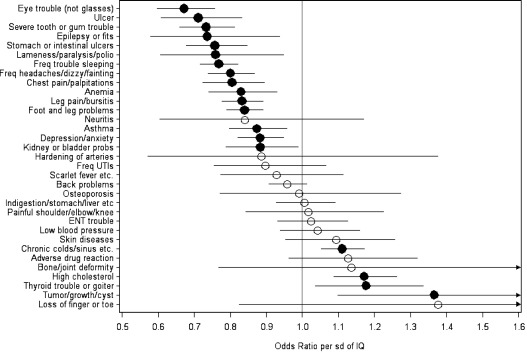
Odds ratios for a self-reported health problem per SD of AFQT score – age adjusted. Filled circles indicate odds ratios significantly different from 1.

Fig. 2 shows the comparable results for the self-reported health problems and the numerical results are shown in Table 3. Higher AFQT score is associated with significantly lower odds of having the problem for 15 out of the 33 and higher odds for four. In most cases, adjusting for background SES attenuates the odds somewhat and those for ‘kidney or bladder problems’ and ‘tumor/growth/cyst’ are no longer significant. Odds ratios range from 0.67 for eye problems, i.e. 33% lower odds of having such problems for one SD advantage in AFQT score, through 0.88 (12% lower odds) for depression/anxiety, to 1.17 for high cholesterol (17% greater odds), and 1.37 (37% higher odds) for tumor/growth/cyst.

## 4 Discussion

Cognitive ability assessed with the AFQT in youth is significantly associated with a wide variety of physical and mental health outcomes at 40 years of age. Overall, those with higher mental ability tended to report better health. The health outcomes were ascertained using various methods: from established scales—such as the CES-D and SF12—through medically diagnosed conditions to self-reported problems. The range of health conditions and problems includes both psychological and somatic health and the latter encompasses numerous bodily systems. Whilst 40 years of age is still relatively young for the major causes of death (e.g. cardiovascular and respiratory disease) a relationship with intelligence is already evident. The results have a number of implications for cognitive epidemiology, in particular, and epidemiology more widely.

First, they suggest that the associations observed between intelligence test scores and later mortality are likely to be mirrored by similar associations with morbidity in mid and later life. Although unsurprising, this nonetheless gives support to the evidence from mortality. In particular, the significant associations with diagnosed hypertension and self-reported chest pain agree with studies showing an association of pre-morbid intelligence with cardiovascular morbidity and mortality and with their risk factors. The exception is the inverse association with self-reported “high cholesterol”. The results for the CES-D score and self-reported depression/anxiety accord with previous studies (Batty et al., 2005; Walker et al., 2002; Zammit et al., 2004) but that for diagnosed “emotional, nervous, or psychiatric problems” does not.

Second, the results indicate that the effect of intelligence extends more widely, for example to respiratory disease (diagnosed chronic lung disease); musculoskeletal problems (diagnosed arthritis/rheumatism; self reported ‘leg pain/bursitis’ and ‘foot and leg problems’) and to other, miscellaneous, self reported problems (e.g. anemia, asthma, ulcers, teeth and eye problems). The result for respiratory disease mirrors the finding of Richards, Strachan, Hardy, Kuh and Wadsworth (2005) that cognitive ability at 15 years was related to lung function at 43 years. The associations with frequent headaches and trouble sleeping are ambiguous as those may be symptoms of somatic illness or more evidence of a link with anxiety and depression. The association with epilepsy is well established (Dodrill & Wilensky, 1990) even to the extent that the different subtypes may be distinguished by the degree of intellectual impairment (Nolan et al., 2003), but in this case it is more likely either that epilepsy has a negative impact on cognitive test scores or that both share a common cause.

Third, the association of intelligence with diverse health outcomes together with its pervasive influence on other important aspects of adult life (Gottfredson, 2004; Herrnstein & Murray, 1994) suggests that its effect on health may also be pervasive and act through a variety of mechanisms. Whalley and Deary (2001) suggest four types of mechanism by which intelligence could affect health and longevity: as a record of bodily insults; as an indicator of system integrity; as a predictor of healthy behaviours; and as a predictor of entry to safer environments. The link with musculoskeletal problems could be an example of the latter, with individuals having lower mental test scores being more likely to be involved in heavy manual work. Cardiovascular disease is related, amongst other things, to health behaviours such as smoking (Batty, Deary, & Macintyre, 2007; Batty, Deary, Schoon et al., 2007; Taylor et al., 2003), alcohol consumption (Batty, Deary et al., 2006, 2008) and diet (Gale, Deary, Schoon, Batty, & Batty, 2007). The link with epilepsy could come under either of the first two mechanisms. At least three of the four types of mechanism have been suggested as possible explanations for the link with psychiatric disorders (Batty et al., 2005; Walker et al., 2002; Zammit et al., 2004). In short, when a broad range of health outcomes is considered, as we do here, the results suggest that multiple mechanisms may well be operating.

This study has a number of notable strengths. The sample is large and population based. Cognitive ability was measured early in adolescence when the effects of morbidity on test scores will have been small. Thus reverse causation bias will be low. The measure of intelligence used, the 1989 revision of the Armed Forces Qualification Test, is highly *g* loaded. Jensen (1980, Table 8.5) estimated the median correlation with other standardized tests at 0.81 – higher than the WAIS and the Stanford Binet. The study has a wide range of outcomes chosen to represent a broad picture of health in mid life and determined independently of any relation to intelligence. The results include statistical control for background socio-economic status.

There are also some weaknesses. Principal amongst these is the fact that the health outcomes are all self-reported. They are, therefore, likely to have lower reliability and validity than more objective measures of health, although the use of established scales (CES-D, SF12) and the inclusion of medically diagnosed conditions will have mitigated this to a certain extent. There is also the possibility of intelligence-related bias in both ascertainment and reporting of health problems. If individuals with higher mental test scores are more likely to undergo routine medical checkups and screening, they would be more likely to have health problems discovered and diagnosed. Greater ‘health literacy’ (Beier & Ackerman, 2003; Gottfredson, 2004) might also enable them to recall and report these problems at a later date. These tendencies would lead to higher rates among those with higher intelligence so that the effects reported here – generally in the opposite direction – would be underestimates.

For some of the outcomes, the study clearly lacks statistical power, either because the outcome is rare at the age of 40 (stroke, congestive heart failure, osteoporosis and hardening of the arteries) or because enquiries concerning its presence were only included 1998 (painful shoulder/elbow/knee, adverse drug reaction, bone/joint deformity and loss of finger/toe). However, while this lack of power is a weakness, discounting the outcomes with low power would reinforce the conclusion of a pervasive, predominantly positive, influence of intelligence. The remaining health problems not associated, either way, with intelligence would then include several where infection plays a large part (Frequent UTIs, Scarlet fever etc., ENT trouble, Chronic colds).

In some cases, it is not clear what the health problems include. The notable example is ‘tumor/growth/cyst’ which could include skin cancer, as this is specifically excluded from the diagnosed cancer. If so, the finding would agree with that of Batty et al. (2007). The fact that the odds ratio for ‘tumor/growth/cyst’ is markedly attenuated by adjusting for parental SES might also suggest this, but it is not possible to be sure. There are also some differences in composition between the baseline sample of the NLSY79 and that analyzed here. Although these are small, the possibility of some attrition bias cannot be ruled out.

Cognitive epidemiology is a thriving, vigorous, youngster showing every sign of a long and productive life to come. This may be as well, because there is still a great deal to explain. The present study has begun to fill the explanatory gap concerning the intelligence-related morbidities that come between the intriguing and important childhood intelligence-later life mortality association.

## Figures and Tables

**Table:** 

Abbreviated label	Full question wording
Stroke	Has a doctor ever told you that you had a stroke?
Congestive heart failure	Has a doctor ever told you that you have congestive heart failure?
Chronic lung disease	Not including asthma, has a doctor ever told you that you have chronic lung disease such as chronic bronchitis or emphysema?
Heart problems	Has a doctor ever told you that you had a heart attack, coronary heart disease, angina, congestive heart failure, or other heart problems?
Hypertension	Has a doctor ever told you that you have high blood pressure or hypertension?
Diabetes	Has a doctor ever told you that you have diabetes or high blood sugar?
Arthritis/rheumatism	Have you ever had, or has a doctor ever told you that you have, arthritis or rheumatism?
	In what month and year was your arthritis or rheumatism diagnosed? (This question was used to exclude reports of arthritis or rheumatism from the previous question that had not been diagnosed.)
Emotional/nervous/psychiatric	Has a doctor ever told you that you had emotional, nervous, or psychiatric problems?
Non-skin cancer	Has a doctor ever told you that you have cancer or malignant tumor of any kind except skin cancer?

**Table:** 

Abbreviated label	Full question wording
Eye trouble (not glasses)	Eye trouble, other than glasses or contacts?
Ulcer	Ulcer?
Severe tooth or gum trouble	Severe tooth or gum trouble?
Epilepsy or fits	Epilepsy or fits?
Stomach or intestinal ulcers	Stomach or intestinal ulcers?
Lameness/paralysis/polio	Lameness or paralysis (including polio)?
Freq trouble sleeping	Frequent trouble sleeping?
Freq headaches/dizzy/fainting	Frequent or severe headaches, dizziness or fainting spells?
Chest pain/palpitations	Pain or pressure in your chest, palpitation or pounding heart, or heart trouble?
Anemia	Anemia?
Leg pain/bursitis	Swollen or painful joints, frequent cramps in your legs or bursitis? (arthritis and rheumatism already addressed)
Foot and leg problems	Problems with your feet and legs?
Neuritis	Neuritis?
Asthma	Asthma? (Shortness of breath or chronic cough?)
Depression/anxiety	Depression or excessive worry or nervous trouble of any kind?
Kidney or bladder probs	Kidney or bladder problems?
Hardening of arteries	Hardening of the arteries?
Freq UTIs	Frequent urinary tract infections? (other than kidney problems discussed earlier)
Scarlet fever etc.	Scarlet fever, rheumatic fever, tuberculosis, jaundice or hepatitis?
Back problems	Problems with your back?
Osteoporosis	Osteoporosis?
Indigestion/stomach/liver etc	Frequent indigestion, stomach, liver or intestinal trouble, gall bladder trouble or gallstones?
Painful shoulder/elbow/knee	Painful or “trick” shoulder or elbow, “trick” or locked knee?
ENT trouble	Ear, nose, or throat trouble?
Low blood pressure	Low blood pressure?
Skin diseases	Skin diseases?
Chronic colds/sinus etc.	Chronic or frequent colds, sinus problems, hay fever or allergies?
Adverse drug reaction	Adverse or allergic reaction to any serum, drug or medicine?
Bone/joint deformity	Bone, joint or other deformity?
High cholesterol	High cholesterol?
Thyroid trouble or goiter	Thyroid trouble or goiter?
Tumor/growth/cyst	Tumor, growth, or cyst? (cancerous or non-cancerous, other than those cancers discussed earlier)
Loss of finger or toe	Loss of finger or toe?

**Table 1:** Regression of CES depression and SF12 physical and mental health scores on AFQT.

	Model							
	Age and sex adjusted				Age, sex and SES adjusted			
Outcome	Beta	SE	*t*	*p*	Beta	SE	*t*	*p*
CES-D score	− 0.186	0.011	− 16.17	< 0.0001	− 0.181	0.014	− 12.99	< 0.0001
SF12 Physical component	0.157	0.012	13.42	< 0.0001	0.125	0.014	8.82	< 0.0001
SF12 Mental component	0.061	0.012	5.19	< 0.0001	0.056	0.014	3.98	< 0.0001

SF12 components are scored so that higher scores indicate better health. Samples sizes: CES-D, 7,458; SF12, 7,423.

**Table 2:** Odds ratio for a diagnosed condition per SD of AFQT score.

Health problem	Age and sex adjusted					Age, sex and SES adjusted		
	Yes	Sample size	Odds ratio	Confidence interval	*p*	Odds ratio	Confidence interval	*p*
Stroke	20	2182	0.66	0.38 to 1.15	0.1470	0.81	0.43 to 1.53	0.5165
Congestive heart failure	20	7458	0.70	0.42 to 1.15	0.1557	0.59	0.32 to 1.07	0.0834
Chronic lung disease	222	7461	0.78	0.67 to 0.90	0.0007	0.77	0.64 to 0.92	0.0037
Heart problems	228	7460	0.84	0.73 to 0.96	0.0136	0.87	0.73 to 1.03	0.1116
Hypertension	1279	7460	0.84	0.79 to 0.89	<.0001	0.89	0.83 to 0.97	0.0047
Diabetes	396	7461	0.88	0.79 to 0.98	0.0168	0.98	0.86 to 1.11	0.7037
Arthritis/rheumatism	797	7454	0.90	0.83 to 0.97	0.0048	0.90	0.82 to 0.99	0.0327
Emotional/nervous/psychiatric	532	7458	1.02	0.93 to 1.12	0.7010	1.02	0.91 to 1.14	0.7251
Non-skin cancer	145	7462	1.08	0.91 to 1.28	0.3752	0.90	0.73 to 1.11	0.3349

The question on Stroke was only included in 2004, hence the smaller sample size.

**Table 3:** Odds ratio for a self reported condition per SD of AFQT score.

Health problem	Age and sex adjusted					Age, sex and SES adjusted		
	Yes	Sample size	Odds ratio	Confidence interval	*p*	Odds ratio	Confidence interval	*p*
Eye trouble (not glasses)	380	7454	0.67	0.60 to 0.76	< .0001	0.72	0.63 to 0.83	< .0001
Ulcer	202	7450	0.71	0.61 to 0.83	< .0001	0.77	0.64 to 0.93	0.0069
Severe tooth or gum trouble	462	7455	0.73	0.66 to 0.81	< .0001	0.77	0.68 to 0.88	< .0001
Epilepsy or fits	84	7452	0.74	0.58 to 0.94	0.0125	0.65	0.49 to 0.87	0.0039
Stomach or intestinal ulcers	396	7450	0.76	0.68 to 0.85	< .0001	0.79	0.69 to 0.90	0.0004
Lameness/paralysis/polio	95	7452	0.76	0.61 to 0.95	0.0153	0.73	0.56 to 0.96	0.0231
Freq trouble sleeping	1191	7453	0.77	0.72 to 0.82	< .0001	0.79	0.73 to 0.86	< .0001
Freq headaches/dizzy/fainting	812	7455	0.80	0.74 to 0.87	< .0001	0.85	0.77 to 0.94	0.0010
Chest pain/palpitations	428	7451	0.80	0.72 to 0.89	<.0001	0.86	0.76 to 0.97	0.0172
Anemia	394	7448	0.83	0.74 to 0.93	0.0013	0.82	0.71 to 0.94	0.0049
Leg pain/bursitis	1107	7455	0.83	0.78 to 0.89	<.0001	0.88	0.81 to 0.95	0.0019
Foot and leg problems	1480	7455	0.84	0.79 to 0.89	<.0001	0.89	0.83 to 0.96	0.0029
Neuritis	40	7439	0.84	0.60 to 1.17	0.3039	0.86	0.58 to 1.28	0.4573
Asthma	580	7448	0.87	0.80 to 0.96	0.0035	0.84	0.75 to 0.94	0.0022
Depression/anxiety	960	7449	0.88	0.82 to 0.95	0.0007	0.88	0.81 to 0.96	0.0053
Kidney or bladder probs	352	7452	0.88	0.79 to 0.99	0.0312	0.92	0.80 to 1.05	0.2274
Hardening of arteries	23	7442	0.89	0.57 to 1.38	0.5915	0.97	0.57 to 1.63	0.8989
Freq UTIs	152	7455	0.90	0.75 to 1.07	0.2173	0.91	0.74 to 1.13	0.3874
Scarlet fever etc.	124	7449	0.93	0.77 to 1.11	0.4220	0.91	0.73 to 1.13	0.3860
Back problems	1820	7452	0.96	0.91 to 1.01	0.1307	0.96	0.90 to 1.02	0.1990
Osteoporosis	68	7447	0.99	0.77 to 1.27	0.9444	1.01	0.75 to 1.38	0.9321
Indigestion/stomach/liver etc	681	7452	1.01	0.93 to 1.09	0.8898	1.02	0.92 to 1.13	0.7081
Painful shoulder/elbow/knee	112	1241	1.02	0.84 to 1.22	0.8689	1.04	0.82 to 1.31	0.7560
ENT trouble	476	7453	1.02	0.93 to 1.13	0.6328	1.02	0.91 to 1.15	0.7575
Low blood pressure	397	7435	1.04	0.94 to 1.16	0.4394	0.94	0.83 to 1.07	0.3615
Skin diseases	207	7455	1.09	0.95 to 1.26	0.2032	1.16	0.98 to 1.37	0.0908
Chronic colds/sinus etc.	1813	7451	1.11	1.05 to 1.17	0.0002	1.08	1.01 to 1.15	0.0267
Adverse drug reaction	176	1240	1.13	0.96 to 1.32	0.1472	1.06	0.88 to 1.29	0.5319
Bone/joint deformity	23	1241	1.14	0.77 to 1.68	0.5254	0.84	0.51 to 1.37	0.4798
High cholesterol	799	7351	1.17	1.09 to 1.26	<.0001	1.26	1.15 to 1.38	<.0001
Thyroid trouble or goiter	266	7448	1.18	1.04 to 1.33	0.0124	1.23	1.05 to 1.43	0.0107
Tumor/growth/cyst	86	1241	1.37	1.10 to 1.70	0.0075	1.27	0.97 to 1.66	0.0862
Loss of finger or toe	13	1241	1.38	0.82 to 2.30	0.2284	1.64	0.87 to 3.07	0.1320

Conditions with *N* ~ 1241 were only included in 1998.

## Appendix A Question wording for health conditions

Question wording for diagnosed health conditions

Question wording for self reported health problems

Do you have any of the following health problems? (other than problems discussed earlier)

## Footnotes

1 Word knowledge and paragraph comprehension are first summed to form a verbal score. The verbal, arithmetic and mathematics scores are then standardised. The AFQT standard score is the sum of arithmetic, mathematics and two times the verbal standard scores and is then converted to a percentile score. The tables used for conversion to standard and percentile scores are given in the document ‘NLSY79 Profiles of American Youth: Addendum to Attachment 106’ which is available as item S09 at http://www.nlsinfo.org/ordering/display_db.php3.
